# Small intestinal bacterial overgrowth in Alzheimer’s disease

**DOI:** 10.1007/s00702-021-02440-x

**Published:** 2021-11-19

**Authors:** Karol Kowalski, Agata Mulak

**Affiliations:** Department of Gastroenterology and Hepatology, Wroclaw Medical University, Borowska 213, 50-556 Wrocław, Poland

**Keywords:** Alzheimer’s disease, Brain–gut–microbiota axis, Calprotectin, Small intestinal bacterial overgrowth

## Abstract

The results of animal studies and clinical data support the gut microbiota contribution to the pathogenesis of Alzheimer’s disease (AD). The aim of this pilot study was to evaluate the prevalence of small intestinal bacterial overgrowth (SIBO) and fecal markers of intestinal inflammation and permeability in AD patients. The study was conducted in 45 AD patients and 27 controls. Data on comorbidities, pharmacotherapy, and gastrointestinal symptoms were acquired from medical records and a questionnaire. SIBO was evaluated using lactulose hydrogen breath test. Fecal calprotectin and zonulin levels were assessed by ELISA assays. The positive result of SIBO breath test was found in 49% of the AD patients and 22% of the controls (*p* = 0.025). The comparative analysis between SIBO-positive and SIBO-negative AD patients with respect to the degree of cognitive impairment, comorbidities and used medications did not reveal any statistically significant difference, except for less common heartburn in SIBO-positive AD patients than in SIBO-negative ones (9 vs 35%, *p* = 0.038). The median fecal calprotectin and zonulin levels in the AD group compared to the control group amounted to 43.1 vs 64.2 µg/g (*p* = 0.846) and 73.5 vs 49.0 ng/ml (*p* = 0.177), respectively. In the AD patients there was no association between the presence of SIBO and fecal calprotectin level. Patients with AD are characterized by higher prevalence of SIBO not associated with increased fecal calprotectin level that may be related to anti-inflammatory effect of cholinergic drugs used in the treatment of AD.

## Introduction

Alzheimer’s disease (AD) is the most common cause of dementia characterized by a progressive decline in cognitive function (Reitz and Mayeux 2014). The main features of the disease are aggregation, oligomerization, and deposition of amyloid beta (Aβ) in the form of plaques as well as formation of neurofibrillary tangles composed of hyperphosphorylated tau protein (Jouanne et al. 2017). Those deposits induce neuroinflammation leading to the synapse loss and neuronal cell death (Köhler et al. 2016). Despite large amount of data on the Aβ role in AD it is still not well known what triggers the amyloid aggregation and plaque formation. Besides the complex biochemical processes involved in the neuronal degeneration, other molecular and neurochemical alterations occur including cholinergic deficit due to basal forebrain degeneration (Martorana et al. 2010). Since acetylcholine is involved in cognitive processes, the so-called cholinergic hypothesis has been proposed according to which an increase in acetylcholine level could restore cognitive deficits (Hampel et al. 2018). In fact, most currently available drug therapies, like acetylcholinesterase inhibitors, are still based on this hypothesis (Ferreira-Vieira et al. 2016). However, the current symptomatic treatment does not cure the disease, therefore, there is a need for new therapeutic strategies targeted at the mechanisms involved in the pathogenesis of AD (Zhu et al.^2020^).

The gut microbiota is a key element in bidirectional interactions between the central nervous system (CNS) and the gastrointestinal tract (Köhler et al. 2016; Dinan and Cryan 2017; Quigley 2017). Recently, disturbances within the brain–gut–microbiota axis, including the CNS and the enteric nervous system (ENS), have been shown to contribute to the pathogenesis of AD (Kowalski and Mulak 2019). The formation of Aβ takes place both in the CNS and the ENS (Puig and Combs 2013; Semar et al. 2013). In addition, a large amount of amyloids is produced by the gut microbiota (Zhao et al. 2015). Bacterial amyloids differ from the CNS amyloids in their primary structure but share similarities in their tertiary structure (Friedland and Chapman 2017). Through molecular mimicry bacterial amyloids may act as prion proteins that induce misfolding of native amyloids leading to their aggregation and deposition in amyloid plaques (Friedland 2015).

The gut microbiota is known to upregulate local and systemic inflammation due to lipopolysaccharides (LPS) from pathogenic bacteria and synthesis of pro-inflammatory cytokines (Lukiw 2016; Zhao et al. 2017; Zhan et al. 2018). The gut dysbiosis may induce increased permeability of the intestinal barrier and the blood–brain barrier further enhancing inflammation at the gut, systemic, and CNS levels (Köhler et al. 2016; Kowalski and Mulak 2019). Alterations in the gut microbiota composition characterized by its decreased diversity and stability contribute to overstimulation of immune system and induction of chronic inflammation observed in the elderly (Jouanne et al. 2017). Age-dependent blood–brain barrier changes, especially the increased permeability in the hippocampus involved in learning and memory processes, allow blood-derived molecules to enter the brain (Zhao et al. 2017). The bacterial overgrowth in the small intestine, where permeability is physiologically higher, might be a source of bacterial products, including amyloids and LPS.

The results of studies in animal models and clinical data support the gut microbiota contribution to the pathogenesis of AD (Köhler et al. 2016; Quigley 2017; Kowalski and Mulak 2019; He et al. 2020; Liu et al. 2020; Sochocka et al. 2019). To the best of our knowledge, until now no data on quantitative microbiota disturbances in AD have been published, although there are some reports in other neurodegenerative diseases such as Parkinson’s disease (Fasano et al. 2013). Similarly, data on intestinal inflammation and permeability markers in AD are limited (Leblhuber et al. 2015, 2017). Results of these studies may allow to determine new therapeutic strategies in AD which encompass influencing intestinal immune system and gut permeability by qualitative and quantitative modifications of microbial composition.

The aim of this study was to evaluate the prevalence of quantitative microbiota disturbances in the form of small intestinal bacterial overgrowth (SIBO) in AD patients. Additionally, markers of intestinal inflammation and permeability, calprotectin, and zonulin levels, respectively, were assessed in stool samples. The analysis of patients’ subgroups with positive and negative breath test results was aimed at evaluating potential relationship between the tested parameters and clinical data including the degree of dementia.

## Materials and methods

### Subjects

The study was conducted in 45 patients (9 M, 36 F) with mild or moderate dementia in the course of AD. All patients were recruited among the subjects hospitalized at the single Alzheimer's Disease Research Center. The degree of cognitive impairment was assessed based on the Mini-Mental State Examination (MMSE). The control group consisted of 27 participants (8 M, 19 F) matched according to age, sex, and body mass index (BMI) (subjects diagnosed with dementia, other mental disorders and CNS diseases were excluded). Data on comorbidities, pharmacotherapy, and gastrointestinal symptoms were acquired from medical records and a questionnaire. The following features were considered as the exclusion criteria: significant comorbidities regarding the CNS disorders and gastrointestinal diseases, previous gastrointestinal surgery except for appendectomy and cholecystectomy, the use of antibiotics or preparation for colonoscopy within the last month prior the stool collection, and the use of nonsteroidal anti-inflammatory drugs except for cardioprotective low-dose acetylsalicylic acid (ASA) (75 mg daily). All subjects were tested for SIBO with hydrogen breath test and were asked to provide a stool sample. The protocol of this study was approved by the local Ethics Committee (KB-491/2017). A written informed consent was obtained from all participants prior to the study enrollment.

### Evaluation of small intestinal bacterial overgrowth (SIBO)

The presence of SIBO was investigated by the use of lactulose hydrogen breath test (LHBT). The concept of breath testing is based on the exhalation of gases (hydrogen) produced solely by the intestinal bacteria following ingestion of a carbohydrate substrate (Bohm et al. 2013). All study participants underwent LHBT after preparation including diet restriction and fasting period of at least 12 h. The test was performed in the fasted state in the morning, after tooth brushing. Food intake, physical exercise, and smoking were not permitted during testing. Exhaled air samples were analyzed using Gastrolyzer^®^ (Bedfont Scientific Ltd, Kent, UK). The air samples were collected at the baseline and then after oral administration of lactulose (10 g in 200 mL of water) at intervals of 15 min during the period of 90 min. An increase above 20 parts per million (ppm) of expired hydrogen after 90 min compared to the basal value indicated a positive result.

### Quantitative evaluation of fecal biomarkers

Stool samples of app. 5 g each were collected according to the instruction. After required preparation the samples were stored at − 20 °C until processing as described previously (Ohlsson et al. 2017). The quantitative evaluations of fecal calprotectin and zonulin were performed by ELISA tests: EK-CAL (Bühlmann Laboratories, Switzerland) and IDK^®^Zonulin (Immundiagnostik AG, Germany), respectively. Noteworthy, very recently, during the current study duration, the accuracy of the used zonulin ELISA assay has been questioned (Ajamian et al. 2019) that is adequately addressed in the discussion.

### Statistical analysis

Due to non-normal distribution of the data, non-parametric statistics were used and results are expressed as median along with the lower and upper quartiles (25Q–75Q). The Mann–Whitney *U* test and chi-squared test were applied to compare differences between the groups. The Spearman’s rank correlation coefficient (*R*) was also calculated to test associations between variables. The value of *p* < 0.05 was considered to be statistically significant.

## Results

### Subjects’ characteristics

In the AD patients the median age was 74 years (57–88), median BMI amounted to 26.9 kg/m^2^, and women constitute 80% of the group. There were no statistically significant differences between the above characteristics compared to the control subjects (Table 1). The median MMSE score in the AD patients amounted to 17.0 (14.5–21.5). The most common comorbidities observed in the AD patients as well as in the controls included arterial hypertension (64 vs 63%, *p* = 0.899), ischemic heart disease (29 vs 7%, *p* = 0.029), hyperlipidemia (29 vs 7%, *p* = 0.029), and type 2 diabetes (24 vs 33%, *p* = 0.414). Mental disorders were present only in the AD group. Among the drugs used only by the AD patients were acetylcholinesterase inhibitors, memantine, quetiapine and others neuroleptics, anticonvulsants, and antidepressants. Moreover, the AD patients more frequently used ASA in the cardioprotective dose of 75 mg daily compared to the controls (*p* = 0.002). There were no other significant differences between the groups regarding pharmacotherapy. The prevalence of gastrointestinal symptoms reported by the AD patients and the controls is presented in Table 2. Constipation (stool consistency described as type 1 or 2 according to the Bristol Stool Form Scale), although common in both groups, was less prevalent in the AD patients (31 vs 62%, *p* = 0.012). Moreover, the AD patients compared to the control subjects less frequently reported heartburn (22 vs 46%, *p* = 0.036). However, there was no significant difference between the groups with respect to proton pump inhibitor (PPI) use (16 vs 19%, *p* = 0.744).

**Table 1:** Basic characteristics of the recruited AD patients and the controls

	AD patients *n* = 45	Controls *n* = 27	*p*	SIBO + AD patients *n* = 22	SIBO– AD patients *n* = 23	*p*
Age range (years) M (25Q–75Q)	57–88 74.0 (68.0–80.0)	60–93 72.0 (68–76)	0.314^a^	58–88 75.0 (67.8–75.0)	57–85 73.0 (68.0–78.0)	0.313^a^
Males/females	9/36	8/19	0.352^b^	3/19	6/17	0.297^b^
BMI (kg/m^2^) M (25Q–75Q)	26.9 (23.9–29.8)	27.8 (24.6–30.4)	0.412^a^	27.0 (24.8–29.8)	26.9 (23.7–29.6)	0.733^a^
MMSE (points) M (25Q–75Q)	17.0 (14.5–21.5)	–	–	16.0 (14.0–20.0)	17.0 (14.5–21.5)	0.723^a^

^a^Mann–Whitney *U* test

^b^*χ*^2^ test

*BMI* body mass index, *M* median, *25Q–75Q* lower and upper quartiles, *MMSE* Mini-Mental State Examination, *SIBO* small intestinal bacterial overgrowth

**Table 2:** The most common gastrointestinal symptoms reported by the AD patients (*n* = 45), the controls (*n* = 26), SIBO-positive AD patients (*n* = 22), and SIBO-negative AD patients (*n* = 23)

Gastrointestinal symptoms	AD patients *n* (%)	Controls *n* (%)	*p*	SIBO+ AD patients *n* (%)	SIBO– AD patients *n* (%)	*p*
Constipation	14 (31)	16 (62)	**0.012**	6 (27)	10 (43)	0.605
Diarrhea	1 (2)	0	0.444	0	1 (4)	0.915
Disturbed defecation	14 (31)	13 (50)	0.114	6 (27)	8 (35)	0.586
Abdominal pain	10 (22)	8 (31)	0.425	5 (23)	5 (22)	0.936
Abdominal bloating	10 (22)	8 (31)	0.425	4 (18)	6 (26)	0.524
Heartburn	10 (22)	12 (46)	**0.036**	2 (9)	8 (35)	**0.038**

*χ*^2^ test, *p *< 0.05 marked in bold

### Breath test results

**Fig. 1:**
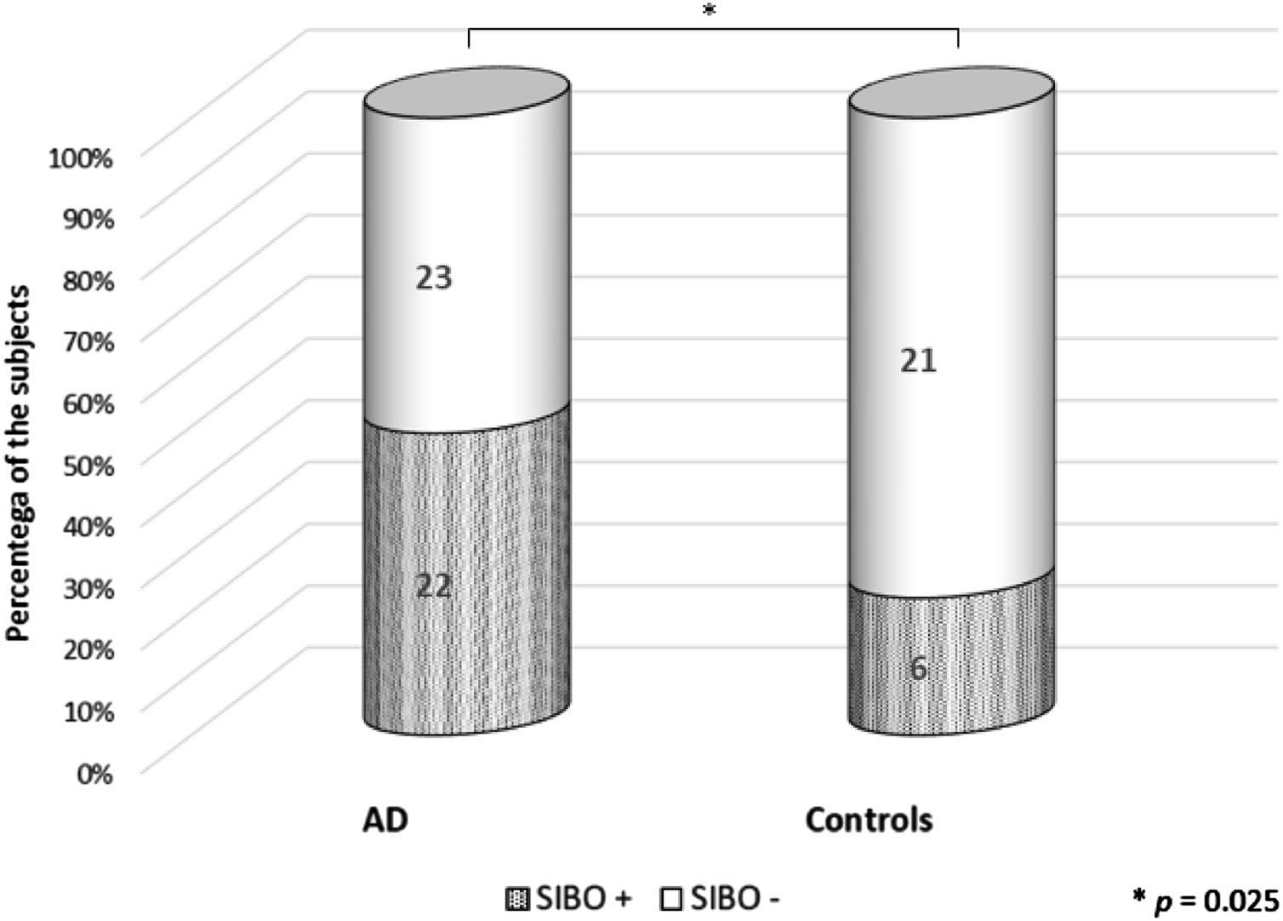
Lactulose hydrogen breath test results in the AD patients and the controls. The prevalence of SIBO was significantly higher in the AD patients than in age-matched controls without dementia (49 vs 22%, *p* = 0.025). The *p* value was calculated by the *χ*^2^ test. *AD* Alzheimer’s disease

The positive result of SIBO breath test was found in 49% of the AD patients and 22% of the control subjects (*p* = 0.025) (Fig. 1). The percentage of women in SIBO-positive and SIBO-negative AD patients was similar (86 vs 74%, *p* = 0.297). Comparing SIBO-positive AD patients (*n* = 22) to SIBO-negative AD patients (*n* = 23) no significant differences in age, BMI, and the MMSE score between these subgroups were found (Table 1). The comparative analysis between those subgroups with respect to comorbidities and used medications did not reveal any statistically significant difference, except for less common heartburn in SIBO-positive AD patients than in SIBO-negative ones (9 vs 35%, *p* = 0.038). Again, these subgroups did not differ with respect to PPI use (14 vs 17%, *p* = 0.728).

### Fecal biomarkers

**Fig. 2:**
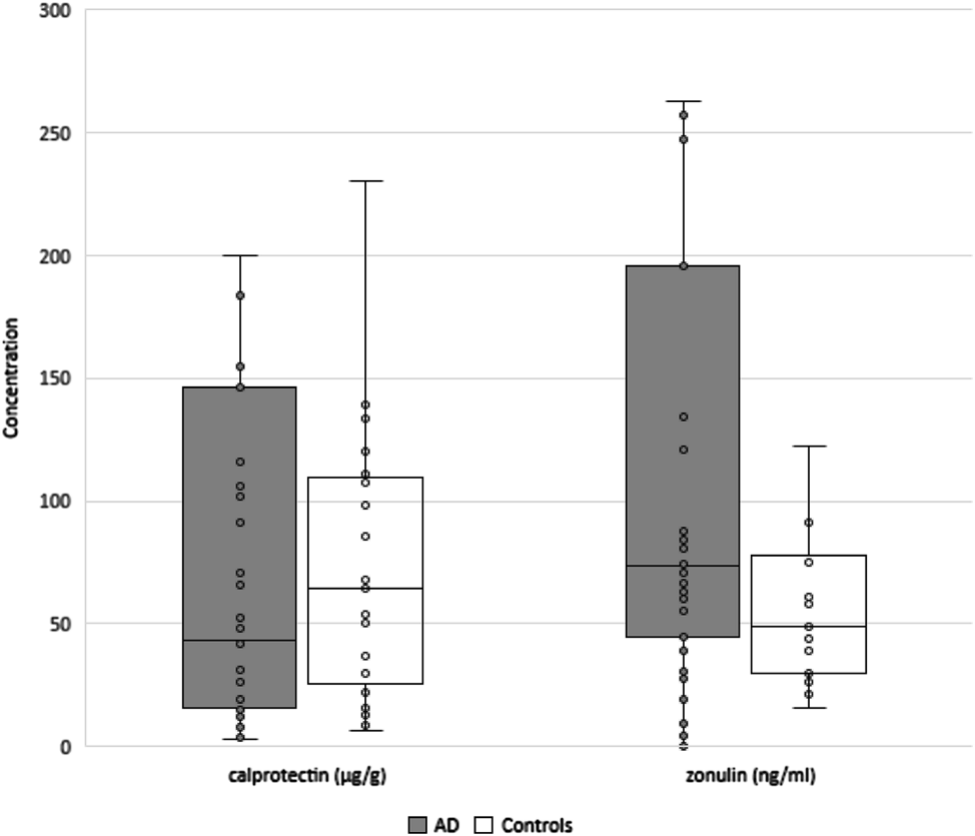
Comparison of fecal calprotectin and zonulin levels in the AD patients and the controls. The median fecal calprotectin and zonulin levels in the AD group compared to the control group amounted to 43.1 vs 64.2 µg/g (*p* = 0.846) and 73.5 vs 49.0 ng/ml (*p* = 0.177), respectively. Individual data are presented excluding outliers. The *p* value was calculated by the Mann–Whitney *U* test. AD, Alzheimer’s disease

The stool samples for quantitative evaluations of fecal calprotectin and zonulin were obtained from 35 AD patients and 25 controls. Calprotectin was evaluated in all control subjects, while zonulin only in 16 of them due to aforementioned controversy regarding the accuracy of the zonulin ELISA assay. The median fecal calprotectin and zonulin levels in the AD group compared to the control group amounted to 43.1 vs 64.2 µg/g (*p* = 0.846) and 73.5 vs 49.0 ng/ml (*p* = 0.177), respectively (Fig. 2). The normal fecal calprotectin level < 50 µg/g was found in 19 AD patients (54%) and 10 control subjects (40%). The numbers of subjects in the above groups with calprotectin levels exceeding 50 µg/g, 112 µg/g and 150 µg/g are presented in Table 3.

**Table 3:** The comparison of fecal calprotectin levels in the AD patients (*n* = 35) and the controls (*n* = 25)

Calprotectin (µg/g)	AD patients *n* (%)	Controls *n* (%)	*p*
< 50	19 (54)	10 (40)	0.275
≥ 50	16 (46)	15 (60)	
< 112	26 (74)	20 (80)	0.606
≥ 112	9 (26)	5 (20)	
< 150	27 (77)	23 (92)	0.128
≥ 150	8 (23)	2 (8)	

*χ*^2^ test

Comparing SIBO-positive AD patients to SIBO-negative AD patients, the median calprotectin and zonulin levels amounted to 52.6 vs 42.2 µg/g (*p* = 0.590) and 63.0 vs 83.8 ng/ml (*p* = 0.568), respectively. There was no correlation between the MMSE score and the level of calprotectin (*R* = 0.22, *p* = 0.306) and zonulin (*R* = 0.05, *p* = 0.808). There was no association between calprotectin and zonulin levels and age, BMI, medication use, and gastrointestinal symptom frequency. Among the AD patients, the calprotectin level was significantly higher in those diagnosed with ischemic heart disease (*p* = 0.017), but no other differences regarding comorbidities were found between the subgroups.

## Discussion

Given the growing understanding of the role of gut microbiota alterations, the gut immune system activation and increased intestinal permeability in AD, the main aim of the study was to evaluate the prevalence of SIBO in the course of AD and its potential association with fecal calprotectin and zonulin levels. The results of this pilot study showed more common prevalence of SIBO in AD patients compared to the matched controls (49 vs 22%, *p* = 0.025). No statistically significant differences were found comparing fecal biomarker levels between the AD patients and the controls as well as between the AD patients with positive and negative LHBT results. Although recently numerous studies have been published on the alterations in the gut microbiota composition in AD (Kowalski and Mulak 2019; He et al. 2020; Liu et al. 2020; Sochocka et al. 2019), to the best of our knowledge, this is the first report on SIBO in AD patients, not including our own preliminary study presented in the abstract form (Kowalski et al. 2017).

Regarding the characteristics of the subjects, 80% of the studied AD patients were women which is consistent with the female predominance in AD (Reitz and Mayeux 2014; Oveisgharan et al. 2018). That may be related to longer life expectancy in women, since age is the main risk factor for dementia (Tom et al. 2015). The most common comorbidities observed in the AD patients included cardiovascular diseases such as arterial hypertension, ischemic heart disease, hyperlipidemia, and type 2 diabetes. A large number of the AD patients were overweight (45%) or obese (20%). All of the above conditions constitute the risk factors for AD (Armstrong 2019). The control subjects, although less numerous, were matched for sex, age, and BMI. Among concomitant diseases ischemic heart disease and hyperlipidemia were more common in the AD patients, and those factors were linked with AD in previous research (Armstrong 2019). Moreover, side effects of the medications used in AD include gastrointestinal symptoms. Cholinergic drugs may induce nausea, vomiting, diarrhea or abdominal pain (Colovic et al. 2013), while the use of memantine may result in constipation (Mimica and Presecki 2009). In the studied groups, the significant differences between the AD patients and the controls concerning the frequency of reported gastrointestinal symptoms were less common constipation and heartburn in the AD patients that could potentially be related to a prokinetic effect of cholinergic drugs. As mentioned above there was no difference between the groups regarding the use of PPIs. The fact that the AD patients more frequently than the controls used cardioprotective dose of ASA could be associated with concomitant ischemic heart disease (29 vs 7%, *p* = 0.029). Nonsteroidal anti-inflammatory drugs can induce an increase in fecal calprotectin level; however, the results of studies regarding this effect with respect to low ASA doses are not consistent (Montalto et al. 2013).

SIBO, assessed by LHBT, was prevalent almost in half of the studied AD patients (49%) compared to 22% of the controls (*p* = 0.025). The available data on the SIBO prevalence in the general population or in the course of different disorders are not fully consistent (Quigley 2019). Moreover, some methodological differences may also affect the results. For comparison, in patients with Parkinson’s disease (PD) SIBO was reported in 25.3% (Tan et al. 2014), 30.2% (Niu et al. 2016), 54.2% (Gabrielli et al. 2011), and 54.5% (Fasano et al. 2013) of the subjects. At the same time in the controls included in the above studies SIBO was found in 9.5% (Niu et al. 2016), 8.3% (Gabrielli et al. 2011), and 20.0% (Fasano et al. 2013). The relatively high percentage of SIBO-positive controls in the current study (22%) may be associated with more advanced age of the subjects. In general, the majority of studies indicate the age-related increase in the SIBO prevalence (Elphick et al. 2005; Parlesak et al. 2003; Choung et al. 2011), although some of them confirm such a relationship only in women (Newberry et al. 2016). Despite the fact that some conditions like type 2 diabetes or chronic PPI use are well-known risk factors for the development of SIBO, we did not observe such a relation in the current study, probably due to limited number of the subjects. The typical symptoms of SIBO include bloating, flatulence, abdominal pain, diarrhea or constipation. Nevertheless, SIBO is a heterogenous syndrome which may stay asymptomatic in otherwise healthy subjects (Bures et al. 2010; Almeida et al. 2008). In the present study, SIBO in AD patients was not associated with a higher prevalence of bowel symptoms, similarly to the earlier research conducted in PD patients (Tan et al. 2014). Interestingly, SIBO-positive AD patients were characterized by less common heartburn compared to SIBO-negative AD patients (9 vs 35%, *p* = 0.038). Simultaneously, the use of PPI in the both subgroups was comparable (14 vs 17%, *p* = 0.728). Presumably, relatively smaller gastric acid secretion protects from heartburn, but predisposes to SIBO. Moreover, in the studied AD patients, no association between the presence of SIBO and the MMSE score was observed. Interestingly, probiotic supplementation in AD patients have been demonstrated to improve cognitive function assessed by the MMSE (Akbari et al. 2016; Tamtaji et al. 2019).

Analyzing fecal calprotectin level as a maker of gut inflammation, no statistically significant differences were found between the AD patients and the controls. Actually, the increased fecal calprotectin level (≥ 50 µg/g) was found in 46 vs 60% of the subjects, receptively (*p* = 0.275). Such a high rate of abnormal results in both groups might be surprising. However, the advanced age of the studied populations has to be taken into consideration. Accordingly to the report by Joshi et al. (2010) the age-related cut-off value of 112 μg/g (instead of 50 µg/g) for fecal calprotectin should be applied in subjects above 60 years of age. In the present study, the fecal calprotectin level was < 112 μg/g in 74% of the AD patients and 80% of the controls. The fecal calprotectin level exceeded 150 μg/g in 8 AD patients (23%) and only in two control subject, but this difference did not reach statistical significance. Fecal calprotectin as a marker of the gut immune system activation is composed of S100A8 and S100A9 proteins. Due to the intrinsically amyloidogenic amino acid sequences of these proteins, they can form amyloid oligomers and fibrils closely resembling amyloid polypeptides such as α-syn and amyloid β (Kowalski and Mulak 2019). Therefore, the intestinal calprotectin pool could contribute to amyloid formation both in the ENS and the CNS. Indeed, the increased S100A9 level in the cerebrospinal fluid in AD patients was found (Horvath et al. 2016), while in one earlier study the increased level of fecal calprotectin in AD patients was also reported (Leblhuber et al. 2015). Moreover, elevated fecal calprotectin level has been observed in PD patients too (Schwiertz et al. 2018; Mulak et al. 2019).

Analyzing the relation between fecal calprotectin level and SIBO presence as well as other clinical characteristics of the AD patients no significant associations were found. Although alterations in the gut microbiota composition may be directly connected with the gut immune system activation and disturbed intestinal barrier function (Musa et al. 2019), there are no clear data on the association between SIBO and fecal calprotectin level. Montalto et al. (2008) showed that patients with SIBO are not characterized with a higher fecal calprotectin level, whereas in patients with concomitant Crohn’s disease (Ricci et al. 2018) or systemic sclerosis (Marie et al. 2015) SIBO was related to increased fecal calprotectin concentration. In the current study no correlation between fecal calprotectin level and the MMSE score in AD was found that is in line with the earlier report by Leblhuber et al. (2015)

Owing to the fact that all recruited patients were already treated for AD, the influence of AD medications on gastrointestinal functions and intestinal inflammation needs to be considered while interpreting the results. Indeed, anti-inflammatory effect of cholinergic drugs has been shown in animal models of colitis (Pai and Yu 2020). Rivastigmine, an acetylcholinesterase inhibitor, alleviates experimentally induced colitis in rodents by acting at central and peripheral sites to modulate immune responses (Shifrin et al. 2013). Also galantamine induces anti-inflammatory effect in a rat model of colitis involving alpha-7 nicotinic acetylcholine receptor (α7nAChR) to suppress pro-inflammatory cytokines (Wazea et al. 2018). In another murine model of colitis, pyridostigmine bromide has been shown to promote mucin synthesis, suppress Th2-dependent inflammation, and attenuate dysbiosis (Singh et al 2020). The above-mentioned effects of the drugs used in the treatment of AD are directly related to the concept of cholinergic anti-inflammatory pathway that is an efferent vagus nerve-based mechanism which regulates immune responses and cytokine production through α7nAChR signaling (Ji et al. 2014; Bonaz et al. 2016). Interestingly, also memantine acting by blocking N-methyl-D-aspartate (NMDA) receptors significantly attenuates macroscopic and microscopic signs of colitis in a mouse model decreasing the plasma levels of interleukin-1β, interleukin-6, and colon level of tumor necrosis factor-α and myeloperoxidase (Motaghi et al. 2016). A potential influence of pharmacotherapy used for AD on investigated fecal markers of gut inflammation and intestinal permeability could result in detecting no significant differences between the AD patients and the controls. To elucidate the role of gut inflammation in the pathogenesis of AD, these markers should be evaluated in de novo diagnosed patients before initiating the treatment.

Zonulin known as a protein regulating intestinal permeability by the modulation of tight junctions has been used so far as a marker of intestinal integrity and permeability (Fasano 2012). Only recently, one of the commercially available ‘zonulin’ ELISA assay, also used in the present study (IDK^®^Zonulin, Immundiagnostik AG, Germany), has been verified to detect complement C3 instead of the actual protein, prehaptoglobin-2 (Ajamian et al. 2019). A potential role of complement C3 in the regulation of the gut barrier function remains unclear. It has also been suggested that complement C3 might be an unspecific product overshadowing the real targets detected by the ELISA assays including one or more members of the zonulin family that have not been discovered yet (Fasano 2020). Due to the methodological limitation of the used zonulin ELISA test, although we decided to present the obtained data, we refrain from the conclusive interpretation. 

Apart from methodological limitations related to fecal zonulin evaluation, other constraints of this preliminary study include relatively small number of the recruited subjects, as well as the fact that all AD patients were already treated. Therefore, the influence of AD medications such as cholinergic drugs on gastrointestinal functions and intestinal inflammation constitutes an important confounding factor. Another weakness of the study is the assessment of SIBO itself, since the accuracy of the used LHBT with a reported specificity of 86% and sensitivity of 52% remains limited (Tan et al. 2014; Bures et al. 2010; Khoshini et al. 2008; Quigley and Abu-Shanab 2010). Nevertheless, this test is still the most widely used technique. The evaluation of SIBO based on the colony counts in the duodenal aspirates is invasive and has also other drawbacks (Bohm et al. 2013). Despite the above shortcomings, this study presents novel data on SIBO presence in AD patients evaluated concomitantly with fecal calprotectin.

In conclusion, the prevalence of SIBO was significantly higher in the AD patients than in age-matched controls without dementia (49 vs 22%). There was no statistically significant difference in fecal calprotectin level in the AD patients compared to the controls which may result from anti-inflammatory effect of cholinergic drugs used in the treatment of AD. In the AD patients, there was no association between the presence of SIBO and fecal calprotectin level, nor the MMSE score. Moreover, no correlation between fecal calprotectin level and the MMSE score in AD was found. Further studies including interventional trials in patients with newly diagnosed AD should help to unravel a causal association between SIBO, gut inflammation and neurodegenerative processes.

## Data Availability

The datasets generated during the current study are available upon request.

## Code availability

N/A.
